# Correlation of EDLC Capacitance with Physical Properties of Polyethylene Terephthalate Added Pitch-Based Activated Carbon

**DOI:** 10.3390/molecules27041454

**Published:** 2022-02-21

**Authors:** Cheol Hwan Kwak, Dohwan Kim, Byong Chol Bai

**Affiliations:** 1 Institute of Carbon Fusion Technology (InCFT), Chungnam National University, Daejeon 34134, Korea; kwakch@cnu.ac.kr; 2 Strategic Planning Division, Korea Institute of Convergence Textile, Iksan 54588, Korea; sunriselloll@gmail.com; 3 Division of Energy Engineering, Daejin University, Pocheon 11159, Korea

**Keywords:** petroleum residue, pitch, PET, activated carbon, EDLC, capacitance

## Abstract

The electric double-layer capacitor (EDLC) has attracted attention by using activated carbon (AC) as an active electrode material with a high power density and high cost-efficiency in industrial applications. The EDLC has been actively developed over the past decade to improve the power density and capacitance. Extensive studies on EDLCs have been conducted to investigate the relation of EDLC capacitance to the physical properties of AC, such as the specific surface area, pore type and size, and electrical conductivity. In this study, EDLC was fabricated with AC, and its capacitance was evaluated with the physical properties of AC. The AC was prepared using petroleum-based pitch synthesized using pyrolysis fuel oil (PFO) with polyethylene terephthalate (PET). The AC based on PFO and PET (PPAC) exhibited high specific surface area and low micropore fraction compared to the PFO-based AC without PET addition (PAC). Furthermore, the reduction of the EDLC capacitance of PPAC was smaller than that of PAC, as the scan rate was increased from 5 to 100 mV s−1. It was determined that the minor reduction of capacitance with an increase in the scan rate resulted from the development of 4 nm-sized mesopores in PPAC. In addition, a comprehensive correlation of EDLC capacitance with various physical properties of ACs, such as specific surface area, pore characteristics, and electrical conductivity, was established. Finally, the optimal properties of AC were thereupon derived to improve the EDLC capacitance.

## 1. Introduction

Interest in electric energy storage has recently grown due to mobile electronics and electric vehicles. Consequently, the demand for energy storage materials such as lithium-ion batteries (LIBs) and electric double-layer capacitors (EDLCs) is increasing [1,2,3]. Numerous studies have been conducted in the past 20 years to improve the high-power density, long cycle life, and capacity of EDLCs [4]. An EDLC stores energy by reversible adsorption and desorption of ions at the electrolyte-electrode interface rapidly, showing a high-power density [5]. In other words, it can produce high power in a short period and thus can be utilized in various electronics such as mobile phones, camera flashes, and electric vehicles. However, it has low energy density because of its limited surface to store ions [6].

A significantly activated carbon (AC) material is most commonly used as an EDLC electrode material. The AC with high conductivity and specific surface area is therefore required to improve the power density and capacity of EDLCs [5]. Recently, AC was obtained by carbonizing waste from fiberboard (medium density) at 500 °C. Activation was performed using KOH activation agents at 800 °C with various KOH/coke mass ratios. Micropores and mesopores could be obtained with specific surface areas above 1500 m^2^ g^−1^. Specific capacitances ranging over 200 F g^−1^ were obtained for the AC electrode. There are design strategies that can improve the energy density of supercapacitors to improve the capacitance and long cycling stability of EDLC materials. A variety of material combinations allow the high capacitance of materials to be exploited. Porous carbon, such as AC materials, can be wisely selected to improve the operating voltage window and the cell’s capacitance, and the pseudocapacitive material acts as an electrode that does not show redox contribution and low stability [5]. Previous studies have found that an increase in specific surface area to a certain level improves the capacitance of EDLC. Still, an increase in specific surface area beyond that level decreases the capacitance due to a reduction of conductivity by micropores [7]. In addition, the adsorption behavior of ions depends on the size of the pores. For example, it was observed in a previous study that an EDLC based on AC with 2–5 nm pores size showed high capacitance [8,9].

Various studies on the relationship of the capacitance to the specific surface area, the electrical conductivity, and the volume of 2–5 nm size of micropores were already performed [10,11,12,13,14,15,16]. However, studies have not been conducted to analyze the overall relationship between the factors listed above that affect capacitance. The specific surface area, pore characteristics, and electrical conductivity of AC are closely related. More specifically, as micropores develop, the specific surface area increases, and the electrical conductivity decreases [17,18]. Therefore, when comparing the properties of AC and the capacitance of an AC-based EDLC, analyzing only one property does not significantly contribute to research on improving the EDLC properties and industrial applications.

Most commercially available devices that use AC as an electrode material operate at a cell voltage of 2.7 V. Specific capacitances of 100 to 120 F g^−1^ and volumetric capacitances up to 60 F cm^−3^ have been reported [19,20]. Electrodes using an aqueous electrolyte had a limited cell operating voltage of up to 0.9 V [21] and a specific capacitance value of 300 F g^−1^ [22]. Past research has focused on the use of three types of AC monoliths as electrodes in half-cell electrochemical systems. The porosity and surface area were improved due to grain formation during oxidation. The maximum capacitance values obtained were 27.68 F g^−1^, 2.23 F g^−1^, and 1.20 F g^−1^ for acid, vapor-oxidized, and non-oxidized electrodes, respectively [23]. According to the report, an energy density of 1.27 Wh kg^−1^ and a specific capacitance of 100 F g^−1^ were obtained for the graphene-gold nanoparticle composite. In one paper, graphene-based carbon with high porosity was fabricated. The carbon obtained gave rise to a Brunauer-Emmett-Teller (BET) surface area of up to 3290 m^2^ g^−1^ values. The resulting specific capacitances were 174 F g^−1^ and ~ 100 F cm^−3^. The observed gravimetric energy and power density values were 74 Wh kg^−1^ and 338 kW kg^−1^, respectively, and the volume values were 44 Wh L^−1^ and 199 kW L^−1^, respectively [24,25].

In this study, the petroleum pitch was prepared using pyrolysis fuel oil (PFO) with heat treatment using polyethylene terephthalate (PET). To control the characteristics of petroleum pitch, studies on the air-blowing process and synthesis using additives such as polyethylene and polystyrene have been researched [26,27,28]. Air-blowing is a polymerization method by which oxygen is added to form crosslinking sites between molecules in the pitch. Blanco et al. have reported that moderate oxygenation improves mesophase content and thermal properties such as softening point and carbon value [29]. However, since the air blowing process requires a long reaction time of 10 h or more, there is a limit in productivity in a commercial operation. Since PET is synthesized from terephthalic acid and ethylene glycol, it forms chemical substances with oxygen functional groups such as hydroxyl groups and carboxylic acids during thermal decomposition [30,31]. These chemicals promote the polymerization of petroleum pitch, and oxygen also provides crosslinking sites during pitch synthesis [32]. Therefore, pitch synthesis using PET as an additive can achieve similar effects to air-blowing despite a short reaction time.

This study evaluated the specific capacitance of EDLCs fabricated using petroleum pitch-based AC electrodes. To analyze the effect of PET addition to pitch on the specific surface area, pore characteristics, and electrical conductivity of the AC, the correlation of capacitance with these properties of AC was investigated. As a result, the specific surface area and pore volume of AC based on PFO and PET (PPAC) were improved in comparison with those of PFO-based AC (PAC) [26]. It is known that the capacitance is strongly affected by the specific surface area and pore characteristics of AC [33]. In other words, the addition of PET has a significant influence on capacitance. Furthermore, 2–5 nm pores, which significantly affect capacitance, are concerned with changes at different scan rates. However, the electrical conductivity of AC has a negligible effect on the capacitance. Finally, the correlation between the capacitance and four AC factors was analyzed to optimize the pore properties and specific surface area of AC for EDLC production: BET surface area, micropore volume, pore volume at 2–5 nm, and electrical conductivity of AC.

## 2. Results

### Capacitance of EDLC Fabricated with Pitch-Based AC

**Figure 1:**
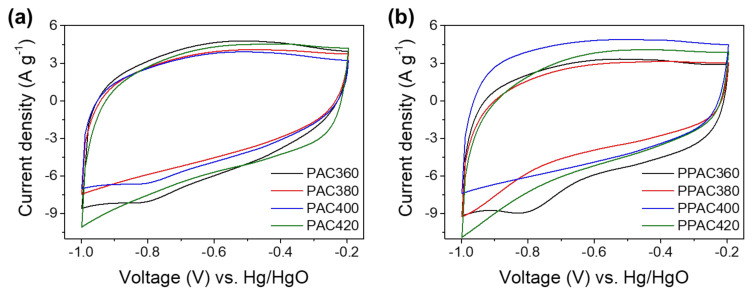
CV curves of EDLC fabricated with electrodes based on (**a**) PAC and (**b**) PPAC prepared by various pitch samples.

EDLC electrodes were manufactured using AC prepared from pitch samples synthesized under various conditions. The pitch was first synthesized using PFO and then activated to prepare AC. The results of measuring the CV curve of EDLC fabricated using these ACs are shown in Figure 1. The CV was measured at a scan rate of 100 mV s^−1^ in a potential window from −1.0 to −0.2 V (vs. Hg/HgO). The CV curves showed a quasi-rectangular shape.

The capacitance (*C*) was calculated by the following equation [34].

(1) $$C=\frac{\int IdV}{M_{e}\times \nu \times \Delta V}$$

here, ∫IdV is the area of the CV curve, *M_e_* is the mass of the active material used, *ν* is the scan rate, and Δ*V* is the potential window. The capacitance is calculated from the CV curve measured at various scan rates in the range of 5–100 mV s^−1^ and is shown in Table 1. It was confirmed that the capacitance of PPAC was about 87.8–96.9% of the capacitance maintained compared to PAC prepared at the same pitch synthesis temperature.

In addition, the retention rate of the capacitance was checked when the scan rate increased. The capacitance retention rate is obtained by calculating the capacitance maintained when the scan rate increases from 5 mV s^−1^ to 100 mV s^−1^. When comparing pitch-based activated carbon synthesized at the same temperature, the PPAC capacitance was 2% higher than the PAC sample. When the activated carbon was manufactured using pitch added with PET, it was confirmed that the change in capacitance according to the scan rate was negligible. This phenomenon was believed to be related to the pore characteristics of the activated carbon, and the pore characteristics of the activated carbon were also analyzed.

## 3. Discussion

### 3.1. Effect of Specific Surface Area and Porosity of AC on EDLC Capacitance

Since the specific surface area, pore characteristics, and conductivity of activated carbon are not independent and influence each other, the influence on the relationship and capacitance of each character should be comprehensively analyzed. Table 2 shows the specific surface area, pore characteristics, and conductivity analysis results of the pitch-based activated carbon prepared in this study. The physical properties of activated carbon changed depending on the synthesis temperature of the pitch used as raw material and the presence or absence of PET. In particular, the specific surface area of PPAC was higher than that of PAC due to adding PET. The changes in the pitch property and specific surface area of activated carbon according to the addition of PET were reported in previous studies [33]. In short, adding PET when synthesizing the pitch using PFO dramatically increases the thermal stability of the pitch. The increase in thermal stability makes the pitch structurally stable and prevents the pores from being formed and closed by intermolecular realignment when the pore structure is developed due to activation. Therefore, although the pitch was synthesized at the same temperature, PPAC using a high-stability PET-added pitch has a higher specific surface area than PAC. However, the microporosity tended to decrease when PET-added pitch was used. This phenomenon is caused by the high structural stability of the pitch interfering with the formation of micropores by the activation reaction. The microporosity showed a distribution of 88.4–90.0% for the PPAC sample, and the microporosity of the PAC sample showed a distribution of 90.3–92.0%.

In general, the increase in the specific surface area and micropore development of activated carbon appear together, and the activated carbon having a high specific surface area has a large micropore volume [35]. The previous study results revealed that the micropores of a size of less than 2 nm have low ion accessibility, and thus the capacitance decreases as the micropore volume increases [36]. Another study reported that pores of a specific size (2–5 nm) were associated with improved capacitance [37]. Several groups have reported low capacitance measurements of activated carbon with large micropore volumes [38,39,40].

**Figure 2:**
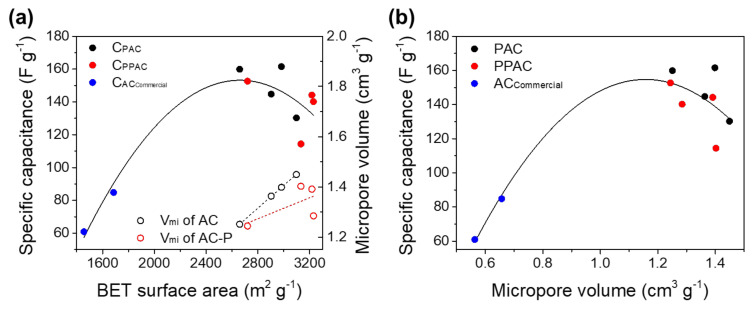
Effect of (**a**) BET surface area and (**b**) micropore volume on the specific capacitance of EDLC prepared by various activated carbon samples.

Figure 2 shows the relationship between the capacitance of the activated carbon electrode and the specific surface area and micropore volume. As shown in Figure 2a, when the specific surface area of the activated carbon increased, the capacitance increased up to about 2600 m^2^ g^−1^ and then decreased. In addition, the micropore volume and specific surface area showed a proportional relationship. Therefore, it is believed that the tendency of the capacitance change is due to the increase in the micropore volume. As the micropore volume increases, the accessible surface area where electrolyte ions can be stored decreases. In addition, as shown in Figure 2b, the tendency of decreasing capacitance due to the increase in micropore volume is similar to the change in capacitance according to the specific surface area of Figure 2a. Therefore, the capacitance decreased when the specific surface area of activated carbon increased because the volume of micropores with low electrolyte ion accessibility increased.

### 3.2. Relationship between Pore Distribution and EDLC Capacitance

**Figure 3:**
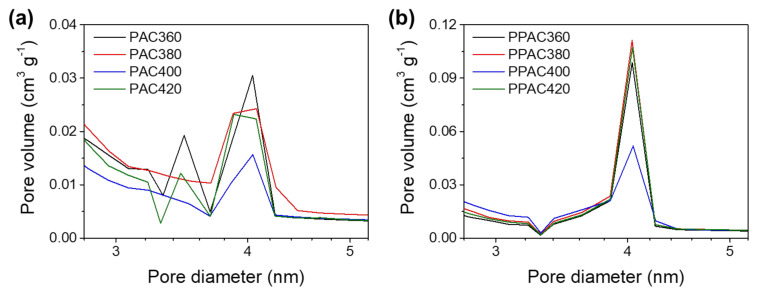
Pore size distribution of (**a**) PAC and (**b**) PPAC.

The PPAC has a higher specific surface area than PAC but low microporosity. However, the PPAC has a lower capacitance than PAC despite the low porosity. Therefore, it is posited that there is a factor that impedes the storage of ions in pores having a size of 2 nm or more. The pore size distributions of PAC and PPAC were compared to analyze this factor. Figure 3 shows the 2–5 nm pore size distribution of PAC and PPAC. All activated carbon had pores distributed around 4 nm, and the PPAC samples developed more pores than PAC. Additionally, PAC360 and PAC420 had pores of about 3.5 and 4 nm in size, unlike other samples.

**Figure 4:**
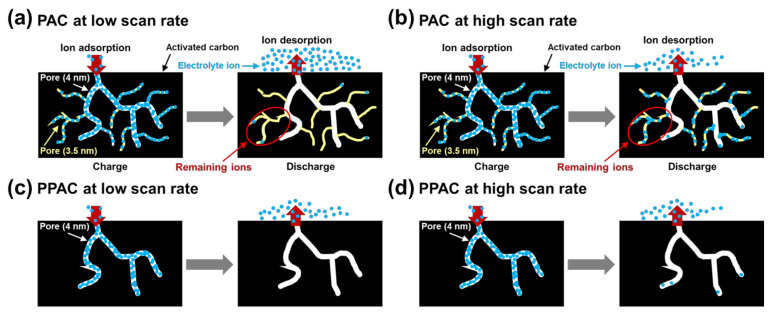
Schematic illustration showing proposed ion storage and expulsion behavior of PAC at (**a**) a low scan rate and (**b**) a high scan rate, and that of PPAC at (**c**) low scan rate and (**d**) high scan rate.

It should be noted here that the capacitances of PAC360 and PAC420 were higher than those of the other samples. When an electric field is applied to the porous electrode, external ions with opposite charges are electrically adsorbed by the pore surface charge and develop an electric double layer. At this time, when the pore size approaches the thickness of the electric double layer, the electric double layer formed by external ions overlaps. If the size of the micropores is smaller than 2 nm, the EDLC properties are lost due to the overlapping of the electric double layer. In addition, according to the results of previous studies, the development of mesopores, especially 2–5 nm pores, improves the capacitance [37]. Table 2 shows the volume of 2–5 nm pores of the activated carbon samples, but it has little correlation with the capacitance measurement. Additionally, the pore volume of 3.5 nm is insignificant compared to the other activated carbon samples. Therefore, the observation that PAC360 and PAC420 have relatively high capacitance is not due to pores of a specific range or a specific size. To explain this phenomenon, a model of pore structure and ion adsorption behavior is proposed in Figure 4. When two or more peaks appear in the pore size distribution curves, such as in the cases of PAC360 and PAC420, it is determined that pores of different sizes are formed separately, but pores of small sizes are formed inside large pores. In this type of pores, ions are stably stored in small-sized pores at low scan rates, but at fast scan rates, they do not move into small-sized pores. They are stored only in large-sized pores, and, consequently, the difference in capacitance according to the scan rate is significant, as shown in Figure 4a. On the other hand, PPAC shows a narrow mesopore distribution, as shown in Figure 4b, but, since the pores are well developed, ions can be stably stored even at a fast scan rate, and thus the capacitance is reduced.

### 3.3. Effect of Conductivity of AC on EDLC Capacitance

Generally, activated carbons show low conductivity among carbon materials because of their various pore structures, such as micropore (~2 nm), mesopore (2~50 nm), and macropore (~50 nm). Microporosity (less than 2 nm) especially acts as a resistance to charge transfer inside the activated carbon particles, and hence the conductivity decreases as the pore develops. In addition, low electrode density due to pore development makes it difficult to increase electrical conductivity when using activated carbon as an electrode material. In particular, improving the specific surface area of activated carbon in the EDLC reduces the conductivity and electrode density as micropores develop, thereby limiting the improvement of capacitance.

**Figure 5:**
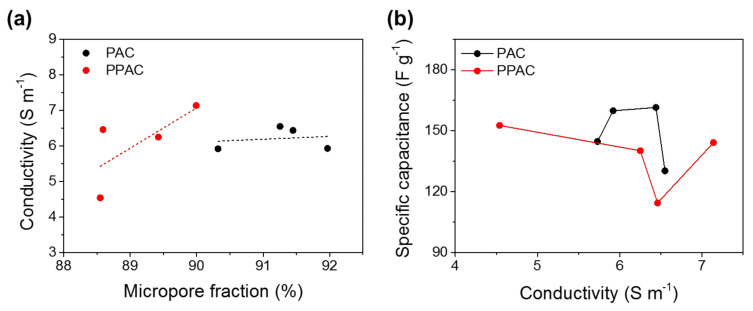
(**a**) Relationship between the electrical conductivity and micropore fraction of activated carbons with different BET surface areas. (**b**) Relationship between the specific capacitance and micropore fraction of activated carbons with different electrical conductivities.

It can be seen from Table 2 that the specific surface area of the pitch-based activated carbon prepared in this study has a high specific surface area of 2500 m^2^ g^−1^ or higher, and micropores are also developed. Therefore, although all samples show low conductivity, PPAC tends to exhibit higher conductivity than PAC. To determine the relationship between the microporosity and conductivity, changes in conductivity with the micropore fraction of ACs were investigated. It is generally known that porous carbon materials tend to increase conductivity when the microporosity reduces [7]. However, the conductivity of PAC and PPAC showed a roughly proportional correlation with the micropore fraction in Figure 5a. This result is considered to be due to the oxygen functional groups. Furthermore, several works of research show that the surface functionality of activated carbon significantly affects electrical conductivity [41,42,43,44]. Therefore, the conductivity of PAC and PPAC was affected by both microporosity and surface functionality, which interrupts to reveal the correlation of electrical conductivity with micropore property. Furthermore, the electrical conductivity was irrelevant to EDLC capacitance due to the influence of surface oxygen functional groups on the electrical conductivity, as shown in Figure 5b.

**Figure 6:**
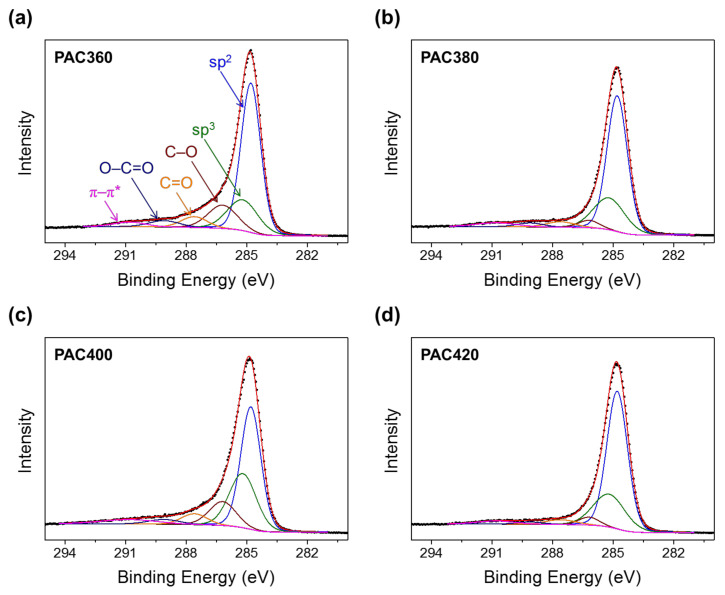
XPS C 1s spectra of (**a**) PAC360, (**b**) PAC380, (**c**) PAC400, and (**d**) PAC420.

In addition, it is observed that PAC360 and PAC400 have relatively high contents of oxygen functional groups in XPS C 1s analysis. Figure 6 shows C 1s spectra of PAC360, PAC380, PAC400, and PAC420 with deconvoluted peaks of sp^2^ (284.8 eV), sp^3^ (285.2 eV), C–O (286.2 eV), C=O (287.6 eV), O–C=O (289.1 eV), and π–π* (290.8 eV) bonds [45]. The sum of the three oxygen functional groups (C–O, C=O, and O–C=O) in PAC360 and PAC400 accounts for 22.4 and 20.5%, respectively, which is higher than that in PAC 380 (9.7%) and PAC420 (8.4%), as shown in Table 3. It should be noted that the electrical conductivity of PAC360 and PAC400 with a high content of surface oxygen functional groups is relatively higher than that of PAC380 and PAC420, but the micropore fraction of all PAC samples is almost the same as 90.3–92.0%. This is proof that the interparticle charge transfer is inhibited due to the surface oxygen functional groups, resulting in the reduction of electrical conductivity in PAC360 and PAC400.

### 3.4. Relationship between Capacitance and Physical Properties of AC

The correlation equation was calculated considering the specific surface area, pore properties, and electrical conductivity of activated carbon to optimize the EDLC capacitance. The equation is indicated below:

(2) $$y=-6.6\times 10^{-4}x_{1}{}^{2}+3.8x_{1}-4.3\times 10^{3}x_{2}{}^{2}+1.1\times 10^{4}x_{2}-4.5\times 10^{6}x_{3}{}^{3}+6.6\times 10^{5}x_{3}{}^{2}-2.8\times 10^{4}x_{3}+30x_{4}-1.3\times 10^{4}$$

where *y* is specific capacitance, *x*_1_ is specific surface area, *x*_2_ is micropore volume, *x*_3_ is mesopore volume at 2–5 nm pore size, and *x*_4_ is electrical conductivity. The coefficient of determination (R^2^) is 0.9976.

The specific surface area and micropore volume are the most influential factors in capacitance. On the other hand, the mesopore volume at 4 nm pore size and electrical conductivity have a negligible effect on the capacitance. In addition, the capacitance has the highest value when *x*_1_ and *x*_2_ are 2879 and 1.28, respectively. In the case of *x*_3_ and *x*_4_, the capacitance proportionally decreases and increases, respectively, with their increase. Meanwhile, *x*_3_ and *x*_4_ have a negligible effect on the capacitance. Therefore, it is found that only 4 nm size mesopores are practical for ion storage. The electrical conductivity is affected by another factor, such as surface functionality. The correlation equation determines that the EDLC capacitance is optimized when the surface area and micropore volumes are 2879 m^2^ g^−1^ and 1.28 cm^3^ g^−1^, respectively.

## 4. Materials and Methods

### 4.1. Materials

PFO (LG Chem, Seoul, Korea) and waste plastic (PET, M.W 48,532, LOTTE Chemical, Seoul, Korea) were used as the raw materials to prepare the pitch. Potassium hydroxide (KOH, 95.0%, Daejung, Siheung-si, Korea) was used as a chemical activating agent to prepare AC. The products of the activation reaction were neutralized by using 35% hydrochloric acid (HCl, Daejung, Siheung-si, Korea) and deionized (DI) water.

### 4.2. Pitch Synthesis Using PFO and PET

For the synthesis of the PFO-based pitch, 500 g of PFO was placed in a 1 L reactor and then heated. In the case of the pitch synthesis using PFO and PET, a mixture of 475 g PFO and 25 g PET was used. A nitrogen atmosphere was maintained in the reactor by supplying nitrogen gas at a flow rate of 200 mL min^−1^. The pitch was synthesized at 360, 380, 400, and 420 °C. The reactor temperature increased to the synthesis temperature from about heating rate of 2.5 °C min^−1^. The reactor was kept for 3 h at the synthesis temperature and then cooled naturally.

### 4.3. Activation of the Pitch Using KOH

KOH’s activating agent was mixed with a 20 g pitch with a blender. The weight ratio of pitch to KOH in the mixture was 1:4. The mixture of pitch and KOH was activated by putting it into an alumina crucible and inserting the crucible into an electric furnace equipped with a SUS tube. The activation was performed at 800 °C for 1 h, while the temperature was increased at a rate of 5 °C min^−1^. A nitrogen atmosphere was maintained in the interior of the SUS tube by injecting nitrogen gas at a flow rate of 100 mL min^−1^. After completing the activation, the reaction products were neutralized using DI water and HCl and then rinsed with DI water. The pore volume was calculated from the N_2_ adsorption-desorption isotherm obtained by the volumetric gas analyzer (3Flex, Micromeritics). The fraction of pore volume was calculated with the pores of less than 3500 Å diameter.

Pitch-based ACs prepared using only PFO synthesized at 360, 380, 400, and 420 °C were denoted as PAC360, PAC380, PAC400, and PAC420. In addition, pitch-based AC prepared by adding PET under the same temperature conditions was also prepared, and the samples were denoted as PPAC360, PPAC380, PPAC400, and PPAC420.

### 4.4. Preparation and Electrochemical Analysis of EDLC

As-prepared ACs and two commercial ACs (YP-50F and YP-80F, Kuraray Co., Ltd., Tokyo, Japan) were used as active materials. The sizes of all AC samples were controlled by less than 20 μm by using a sieve to fabricate the electrode. The active material, carbon black (Super P, Timcal, Bodio, Switzerland) as a conductive material, and polyvinylidene fluoride (PVDF, Sigma-Aldrich, St. Louis, MO, USA) as a binder were mixed in N-methyl-2-pyrrolidone (NMP, Sigma-Aldrich, St. Louis, MO, USA) with a mass ratio of 9:1:1, respectively. A slurry was prepared from the mixture using a Thinky mixer (10 min at 2000 rpm and 10 min at 2200 rpm). This slurry was uniformly coated on a current collector of carbon paper (about 150 μm in thickness) using a doctor blade apparatus (gap 450 μm) and dried in a vacuum oven at 120 °C for 12 h. The mass loading of active materials was measured from 14.8 to 18.0 mg cm^−2^.

The electrochemical analysis of the EDLC based on the AC electrode was carried out in a three-electrode cell. The AC electrode (working electrode) with a 19 mm diameter, the graphite block (counter electrode), and the Hg/HgO reference electrode were equipped in the three-electrode cell filled with the 6 M KOH electrolyte. The three-electrode cell was connected to the potentiostat instrument to analyze the electrochemical property.

### 4.5. Characterization

The nitrogen adsorption and desorption isotherms were obtained using a specific surface area measurement instrument (Micromeritics 3Flex instrument). The specific surface area (S_BET_) and the total pore volume (V_T_) were calculated from the isotherms. The samples were pre-treated in a vacuum oven at 200 °C for 8 h, and the specific surface area was measured at 77.3 K through nitrogen adsorption and desorption. The specific surface area was measured using the multipoint BET method, and the micropore volume (V_mic_) was calculated using the t-plot method. An electrochemical characterization instrument (ZIVE SP2, WonATech, Seoul, Korea) was used for the cyclic voltammetry (CV) analysis of the EDLC. The CV curve was measured in the potential window from −1.0 to −0.2 V, with various scan rates from 5 to 100 mV. The specific capacitance of EDLC was calculated based on the result of the CV curve. The electrical conductivity of the AC powder was measured using a powder resistivity measurement system (HPRM-FA2, Hantech, Ulsan, Korea). A four-electrode cell with 1 cm^2^ was filled with 15 g of AC powder. The resistance of AC powder was measured while applying a pressure of 2000 kgf cm^−2^ to the cell. The conductivity was calculated using the measured resistance and the cell volume.

## 5. Conclusions

It is known that the specific surface area, micropore volume, mesopore volume at 2–5 nm, and electrical conductivity of activated carbon affect the EDLC capacitance. However, a comprehensive relation between these physical properties of activated carbon is still not well established. Therefore, in this study, the correlation between the EDLC capacitance and the physical properties of activated carbon was investigated, and a correlation equation was determined to optimize the specific surface area and micropore volume to enhance the capacitance. According to the correlation equation, the optimized specific surface area and micropore volumes were 2879 m^2^ g^−1^ and 1.28 cm^3^ g^−1^, respectively.

**Figure A1:**
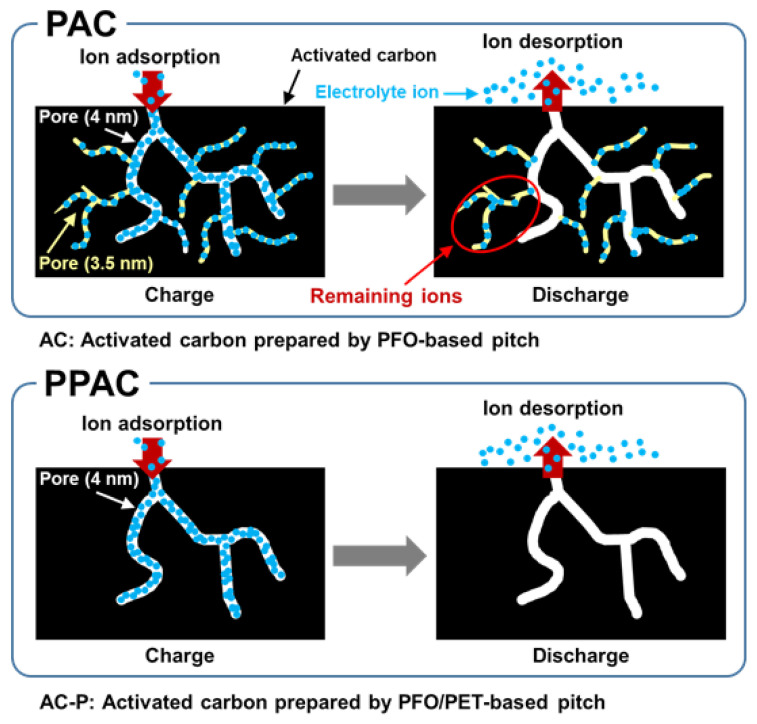
Schematic illustration showing proposed ion storage and expulsion behavior of PAC and PPAC samples.

In addition, activated carbon was prepared using petroleum-based pitch synthesized using PFO with PET addition. It was observed that the EDLC capacitance slightly declined as the scan rate increased from 5 to 100 mV s^−1^ when the electrode was fabricated with PPAC. This result was obtained due to activated carbon’s 4 nm size mesopores. The mesopores at a 4 nm size of PPAC were more developed than PAC. As shown in Figure A1, it is strongly contended that this size of mesopore volume effectively enables ion storage.

This study, therefore, provides insight for the development of EDLCs with high capacitance based on an activated carbon electrode, and the physical properties of the activated carbon are optimized. Furthermore, a mechanism of ion storage based on various pore characteristics is proposed.

## Figures and Tables

**Table 1:** Various specific capacitance at different scan rates from 5 mV s^−1^ to 100 mV s^−1^.

Sample	Specific Capacitance at Different Scan Rate (F g^−1^)					Cν_100_/Cν_5_ ^a^ (%)
	5 mV s^−1^	10 mV s^−1^	20 mV s^−1^	50 mV s^−1^	100 mV s^−1^	
PAC360	280.0	261.4	229.5	191.9	159.9	57.1
PAC380	228.2	211.3	180.8	151.5	130.2	57.1
PAC400	222.0	216.2	191.7	165.2	144.7	65.2
PAC420	303.3	253.1	211.5	290.9	161.5	53.2
PPAC360	247.3	250.7	218.4	176.7	152.6	61.7
PPAC380	193.9	187.4	169.4	141.0	114.4	59.0
PPAC400	200.3	215.2	192.0	162.6	140.2	70.0
PPAC420	255.9	234.2	208.7	173.9	144.2	56.4

^a^ Cν_100_/Cν_5_: Ratio of capacitance at 100 mV s^−1^ to that at 5 mV s^−1^.

**Table 2:** BET analysis results and the electrical conductivity of activated carbon samples.

Sample	Conductivity ^a^ (Sm^−1^)	BET Analysis					Pore Fraction (%)		
		S_BET_ ^b^ (m^2^/g)	V_mi_ ^c^ (cm^3^ g^−1^)	V_t_ ^d^ (cm^3^ g^−1^)	V_2–5 nm_ ^e^ (cm^3^ g^−1^)	M_f_ ^f^ (%)	Micropore	Mesopore	Macropore
PAC360	5.92	2659	1.25	1.38	0.08	90.3	90.3	8.3	1.4
PAC380	6.55	3098	1.38	1.51	0.05	91.3	91.3	7.4	1.3
PAC400	5.93	2903	1.32	1.44	0.02	92.0	92.0	5.2	2.8
PAC420	6.44	2982	1.36	1.48	0.05	91.4	91.4	5.8	2.8
PPAC360	4.54	2720	1.24	1.41	0.06	88.5	88.5	8.1	3.4
PPAC380	6.46	3133	1.35	1.53	0.08	88.6	88.6	8.5	2.9
PPAC400	6.25	3230	1.42	1.59	0.06	89.4	89.4	10.2	0.4
PPAC420	7.14	3217	1.42	1.58	0.05	90.0	90.0	9.0	1.0

^a^ Electrical conductivity at 2000 kgf cm^−2^; ^b^ S_BET_: BET surface area; ^c^ V_mi_: Micropore volume; ^d^ V_t_: Total pore volume; ^e^ V_2–5 nm_: Pore volume with a size of 2–5 nm; ^f^ M_f_: Micropore fraction.

**Table 3:** Position and quantitative analysis of XPS C 1 s peaks.

Bond Component	Binding Energy (eV)	Percentage of Surface Functional Group (%)			
		PAC360	PAC380	PAC400	PAC420
sp^2^ C	284.8	56.5	63.6	47	64.9
sp^3^ C	285.1	18.3	23.2	27.9	23.9
C–O	286.2	13.2	3.6	12.3	3.6
C=O	287.6	5.4	3.6	5.3	3.3
O–C=O	289.1	3.8	2.5	2.9	1.5
π–π*	290.8	2.8	3.6	4.6	2.7
